# Contributions of p38 and ERK to the antinociceptive effects of TGF-β1 in chronic constriction injury-induced neuropathic rats

**DOI:** 10.1186/s10194-016-0665-2

**Published:** 2016-08-19

**Authors:** Nan-Fu Chen, Wu-Fu Chen, Chun-Sung Sung, Ching-Hsiang Lu, Chun-Lin Chen, Han-Chun Hung, Chien-Wei Feng, Chun-Hong Chen, Kuan-Hao Tsui, Hsiao-Mei Kuo, Hui-Min David Wang, Zhi-Hong Wen, Shi-Ying Huang

**Affiliations:** 1 Department of Marine Biotechnology and Resources, National Sun Yat-sen University, #70 Lien-Hai Rd, Kaohsiung, 80424 Taiwan; 2 Division of Neurosurgery, Department of Surgery, Kaohsiung Armed Forces General Hospital, Kaohsiung, 80284 Taiwan; 3 Department of Neurological Surgery, Tri-Service General Hospital, National Defense Medical Center, Taipei, 11490 Taiwan; 4 Department of Neurosurgery, Kaohsiung Chang Gung Memorial Hospital and Chang Gung University College of Medicine, Kaohsiung, 83301 Taiwan; 5 Department of Anesthesiology, Taipei Veterans General Hospital, Taipei, 11217 Taiwan; 6 School of Medicine, National Yang-Ming University, Taipei, 11221 Taiwan; 7 Doctoral Degree Program in Marine Biotechnology, National Sun Yat-sen University and Academia Sinica, Kaohsiung, 80424 Taiwan; 8 Department of Obstetrics and Gynecology, Kaohsiung Veterans General Hospital, Kaohsiung, 81362 Taiwan; 9 Department of Obstetrics and Gynecology and Institute of Clinical Medicine, National Yang-Ming University, Taipei, 11221 Taiwan; 10 Department of Pharmacy and Graduate Institute of Pharmaceutical Technology, Tajen University, Pingtung, 90741 Taiwan; 11 Center for Neuroscience, National Sun Yat-sen University, #70 Lien-Hai Rd, Kaohsiung, 80424 Taiwan; 12 Graduate Institute of Biomedical Engineering, National Chung Hsing University, Taichung, 40227 Taiwan; 13 Center for Stem Cell Research, Kaohsiung Medical University, Kaohsiung, 80708 Taiwan; 14 College of Oceanology and Food Scienece, Quanzhou Normal University, Quanzhou, 362000 China; 15 Marine Biomedical Laboratory and Center for Translational Biopharmaceuticals, Department of Marine Biotechnology and Resources, National Sun Yat-sen University, Kaohsiung, 80424 Taiwan

**Keywords:** Transforming growth factor-β, p38, Extracellular signal-regulated kinase, Chronic constriction injury, Neuropathic pain

## Abstract

**Background:**

Transforming growth factor-βs (TGF-βs) are a group of multifunctional proteins that have neuroprotective roles in various experimental models. We previously reported that intrathecal (i.t.) injections of TGF-β1 significantly inhibit neuropathy-induced thermal hyperalgesia, spinal microglia and astrocyte activation, as well as upregulation of tumor necrosis factor-α. However, additional cellular mechanisms for the antinociceptive effects of TGF-β1, such as the mitogen-activated protein kinase (MAPK) pathway, have not been elucidated. During persistent pain, activation of MAPKs, especially p38 and extracellular signal-regulated kinase (ERK), have crucial roles in the induction and maintenance of pain hypersensitivity, via both nontranscriptional and transcriptional regulation. In the present study, we used a chronic constriction injury (CCI) rat model to explore the role of spinal p38 and ERK in the analgesic effects of TGF-β1.

**Methods:**

We investigated the cellular mechanisms of the antinociceptive effects of i.t. injections of TGF-β1 in CCI induced neuropathic rats by spinal immunohistofluorescence analyses.

**Results:**

The results demonstrated that the antinociceptive effects of TGF-β1 (5 ng) were maintained at greater than 50 % of the maximum possible effect in rats with CCI for at least 6 h after a single i.t. administration. Thus, we further examined these alterations in spinal p38 and ERK from 0.5 to 6 h after i.t. administration of TGF-β1. TGF-β1 significantly attenuated CCI-induced upregulation of phosphorylated p38 (phospho-p38) and phosphorylated ERK (phospho-ERK) expression in the dorsal horn of the lumbar spinal cord. Double immunofluorescence staining illustrated that upregulation of spinal phospho-p38 was localized to neurons, activated microglial cells, and activated astrocytes in rats with CCI. Additionally, increased phospho-ERK occurred in activated microglial cells and activated astrocytes. Furthermore, i.t. administration of TGF-β1 markedly inhibited phospho-p38 upregulation in neurons, microglial cells, and astrocytes. However, i.t. injection of TGF-β1 also reduced phospho-ERK upregulation in microglial cells and astrocytes.

**Conclusions:**

The present results demonstrate that suppressing p38 and ERK activity affects TGF-β1-induced analgesia during neuropathy.

## Background

Globally, 1.5 billion people experience pain [1]. Chronic pain occurs in approximately 20 % of the general population [2, 3], and the prevalence of neuropathic pain has been reported at 6.9 % [3]. Furthermore, drug treatments are not capable of relieving all neuropathic pain conditions [4, 5]. The cellular mechanisms of neuropathic pain are complex and have not been fully elucidated. In 2009, Echeverry et al. reported that intrathecal (i.t.) infusion of transforming growth factor-β1 (TGF-β1) significantly attenuated nerve injury-induced neuropathic pain in rats [6], which suggests two primary research directions. First, the antinociceptive properties of TGF-β1 [7] and its mechanisms [8, 9] must be elucidated. Second, research is required in order to investigate direct involvement of TGF-β1 in the antinociceptive mechanisms of drug compounds [10] or cell-based therapies [9]. However, only a few subsequent studies have investigated the cellular mechanisms of the antinociceptive effects of TGF-β1.

During neuropathy, spinal cord neuroinflammation may promote central sensitization, thereby contributing to the development and maintenance of pain [11, 12]. Spinal neuroinflammation in peripheral neuropathy is characterized by activation of microglia and astrocytes, as well as upregulation of the proinflammatory mediator, tumor necrosis factor-α (TNF-α) [8, 13, 14]. Microglia and astrocytes synthesize TNF-α [15], and TNF-α contributes to neuropathic pain [16, 17]. Additionally, inhibiting activation of microglia and astrocytes [18–20], as well as spinal TNF-α [21] have analgesic effects. Activation of p38 or extracellular signal-regulated kinase (ERK), subgroups of mitogen activated protein kinases (MAPKs), stimulate TNF-α gene expression in primary microglia and astrocytes [15]. Furthermore, peripheral nerve injury and spinal cord injury activate spinal p38 and ERK [22–24]. Several previous studies have suggested that inhibiting p38 [22, 23, 25] and ERK [24] activity are potential therapeutic strategies for neuropathic pain. However, information is limited regarding the roles of p38 and ERK in the antinociceptive effects of TGF-β1 in rat models of neuropathy. In the present study, we examined the effects of i.t. TGF-β1 on p38 and ERK activation in the spinal cord of rats with chronic constriction injury (CCI), a commonly used model of neuropathic pain [26]. We also assessed alterations in the time courses for the antinociceptive effects of TGF-β1 and for activation of p38 and ERK in rats with CCI, in order to further investigate the roles of p38 and ERK in both the development and maintenance of the antinociceptive effects of TGF-β1 during neuropathic pain. We then studied cellular specificity of the effects on p38 and ERK activation in neuropathic rats, including in neurons, microglia, and astrocytes.

## Methods

### Animals

Male Wistar rats (260–285 g) were housed in a temperature- (22 ± 1 °C) and light-cycle-controlled (12 h light/12 h dark) experimental animal house, with free access to food and water. We complied with the Guiding Principles in the Care and Use of Animals of the American Physiology Society and all experiments were approved by the National Sun Yat-sen University and Use Committee. Rats were anesthetized by isoflurane inhalation (2 %) for surgery and drug injections, and all rats received postoperative administration of intramuscular veterin (cefazolin; 0.17 g/kg) in order to prevent infection. The experimental design and procedures aimed to minimize the number of rats used and any distress that they would experience.

### Induction of peripheral mononeuropathy

CCI surgeries were performed on the right sciatic nerve of rats, using the method described by Bennett and Xie [26] and in our previous studies [13, 18]. In brief, we exposed the right sciatic nerve at mid-thigh level, dissected a 5 mm length of nerve, applied four loose ligatures around the sciatic nerve (4-0 chromic gut at 1 mm intervals), and then sutured both the muscle and the skin incision. For the sham-operated group, we exposed the right sciatic nerve but did not perform ligation.

### Implantation of i.t. catheters

We implanted i.t. catheters (PE5 tubes: 9 cm long, 0.008 in. inner diameter, 0.014 in. outer diameter; Spectranetics, Colorado Springs, CO, USA) to the lumbar enlargement of the spinal cord, via the atlanto-occipital membrane at the base of the skull, as previously described by Yaksh and Rudy [27] and our previous studies [13, 18]. For spinal administration, we externalized and fixed an end of the catheter to the cranial side of the rat’s head. The dead volume of the catheters were 3.5 μL. Therefore, an artificial cerebrospinal fluid (CSF) flush (10 μL) followed all i.t. injections, in order to ensure complete delivery of recombinant human TGF-β1 (cat. 100-21; PeproTech, Rocky Hill, NJ, USA) or vehicle. The composition of artificial CSF was (in mM): 122.7 Cl^−^, 151.1 Na^+^, 2.6 K^+^, 1.3 Ca^2+^, 0.9 Mg^2+^, 21.0 HCO_3_^−^, 2.5 HPO_4_^2−^, and 3.5 dextrose, with 5 % CO_2_ in 95 % O_2_ to achieve a final pH of 7.3. Rats were excluded from the study if they exhibited gross neurological injury or had fresh blood in the CSF 5 d after catheter implantation. We also assessed locomotor functioning using the Basso, Beattie, and Bresnahan (BBB) locomotor scale [28], as described previously [13, 18].

### Behavioral testing

We assessed thermal hyperalgesia according to the method described by Hargreaves et al. [29] and our previous studies [30, 31]. In brief, rats were placed in compartmentalized plastic chambers on an elevated glass platform, and hyperalgesia was assessed using an IITC analgesiometer (IITC Inc., Woodland Hills, CA, USA). We used a radiant heat source to target the middle of the plantar surface with low-intensity heat (active intensity = 25) and a cut-off time of 30 s. Paw withdrawal latencies (PWL) were recorded by observing behavioral indications of nociception (withdrawal or licking). We transformed PWL data into a percentage of the maximum possible effect (%MPE) using the following formula: % MPE = ((post-drug latency − baseline)/(cut-off − baseline)) × 100 %. Post-drug latency represents PWL measured after i.t. injection of TGF-β1 or vehicle, and baseline represents PWL measured immediately before i.t. injection.

### Spinal immunohistofluorescence analyses

We mounted the rat lumbar spinal samples from multiple groups into the same OCT block and simultaneously sectioned them with a cryostat at −30 °C (HM550; Microm, Waldorf, Germany) in order to decrease variation in the immunohistochemical processes. Spinal immunohistofluorescence analyses were conducted using a modification of the method described by Sung et al. [32] and our previous studies [18, 31]. For double immunofluorescence staining of phosphorylated p38 (phospho-p38) and either microglia or astrocyte markers, spinal sections (10 μm) were incubated with a mixture of primary antibodies for anti-phospho-p38 (1:100, Thr180/Tyr182, cat. 4511; Cell Signaling Technology Inc., Beverly, MA, USA; monoclonal rabbit antibody) and anti-OX-42 (CD11b, microglia marker, 1:200, cat. CBL1512; EMD Millipore, Temecula, CA, USA; monoclonal mouse antibody) or anti-glial fibrillary acidic protein (GFAP; astrocyte marker, 1:200, cat. MAB3402; EMD Millipore; monoclonal mouse antibody) antibodies, overnight at 4 °C. Spinal section were then incubated with a mixture of Alexa Fluor 488-labeled chicken anti-mouse IgG antibody (1:400, cat. A-21200; Molecular Probes, Eugene, OR, USA; green fluorescence) and DyLight 549-conjugated donkey anti-rabbit IgG antibody (1:400, cat. 711-506-152; Jackson ImmunoResearch Laboratories Inc., West Grove, PA, USA; red fluorescence) for 40 min at room temperature. For double immunofluorescence staining of phosphorylated ERK (phospho-ERK) and microglia or astrocyte markers, spinal sections (10 μm) were incubated with a mixture of primary antibodies, anti-phospho-ERK antibodies (1:100, Thr202/Tyr204, cat. 9101; Cell Signaling Technology Inc.; polyclonal rabbit antibody) and anti-OX-42 (1:200) or anti-GFAP (1:200) antibodies, overnight at 4 °C. Spinal sections were then incubated with a mixture of Alexa Fluor 488-labeled chicken anti-mouse IgG antibody (1:400) and DyLight 549-conjugated donkey anti-rabbit IgG antibody (1:400) for 40 min at room temperature. We used a Leica DM-6000 CS fluorescence microscope (Leica Instruments Inc., Wetzlar, Germany) to visualize the stained spinal sections, recorded images using a SPOT Xplorer Digital camera (Diagnostic Instruments, Inc., Sterling Heights, MI, USA), and then measured the pixel values of the immunoreactive-positive areas using Image J software (National Institutes of Health, Bethesda, MD, USA) with three sections per rat. Spinal neurons distributed over the superficial laminae (laminae I-III) respond to nociceptive stimuli, and these neurons directly contribute to the transmission of nociception [33]. Therefore, the superficial laminae have more crucial roles in neuropathic pain compared to the deep laminae. Thus, we quantified immunoreactivity for the targeted proteins in the superficial laminae, as described by previous studies in rodents with neuropathy [10, 33–35]. Immunofluorescence data is presented as a percentage change compared to the sham operation plus vehicle group, which were regarded as 100 %. Finally, for double immunofluorescence staining for neurons and phospho-p38 or anti-phospho-ERK, spinal sections were incubated with a mixture of anti-neuronal nuclei (NeuN; neuron-specific nuclear protein, 1:500, Alexa Fluor 488 conjugated antibody, cat. MAB377X, EMD Millipore, Temecula, CA, USA; monoclonal mouse antibody) and anti-phospho-p38 (1:100) or anti-phospho-ERK (1:100) antibodies overnight at 4 °C. Spinal sections were then incubated with DyLight 549-conjugated anti-rabbit IgG antibody (1:400) for 40 min at room temperature.

### Data and statistical analyses

All data are represented as means ± standard errors on the mean (SEMs). Between groups differences were calculated using one-way analyses of variance (ANOVAs). Effects were further investigated using Student-Newman-Keuls post hoc tests, with statistical significance set at *P* < 0.05.

## Results

### Effects of i.t. TGF-β1 on CCI-induced nociceptive behavior

**Fig. 1:**
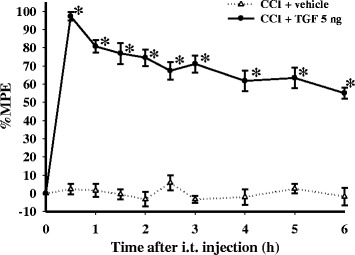
Time course of anti-hyperalgesic effects of transforming growth factor-β1 (TGF-β1) in rats with chronic constriction injury (CCI) at 14 d post-surgery. The x-axis represents the time (h) from intrathecal (i.t.) injection of vehicle or TGF-β1 (5 ng), and the y-axis represents the percentage of the maximum possible effect (%MPE) for anti-hyperalgesia. TGF-β1 significantly attenuated CCI-induced thermal hyperalgesia in rats at 14 d post-surgery. Each point shows the mean ± standard error of the mean (SEM) from six rats per group. *P < 0.05 compared to the CCI plus vehicle group at the same time points

Based on our previous findings [8], we selected a 5 ng dose of TGF-β1 for the present study. At 14 d post-surgery, i.t. injections of vehicle did not significantly affect thermal hyperalgesia in rats with CCI (Fig. 1). Compared to the vehicle group, the anti-hyperalgesic effect of TGF-β1 reached the maximum %MPE at 0.5 h after the i.t. injection, and then decreased gradually over time. The effect persisted at > 50 % MPE for at least 6 h after TGF-β1 administration. In addition, the vehicle- and TGF-β1-treated CCI groups exhibited normal behaviors (including locomotor function). We then focused on three time points (0.5, 3, and 6 h) after i.t. administration of TGF-β1, in order to determine whether modulation of spinal phospho-p38 and phospho-ERK are involved in the antinociceptive effects of TGF-β1 in neuropathic rats.

### Effects of i.t. TGF-β1 on CCI-induced upregulation of spinal phospho-p38 expression

**Fig. 2:**
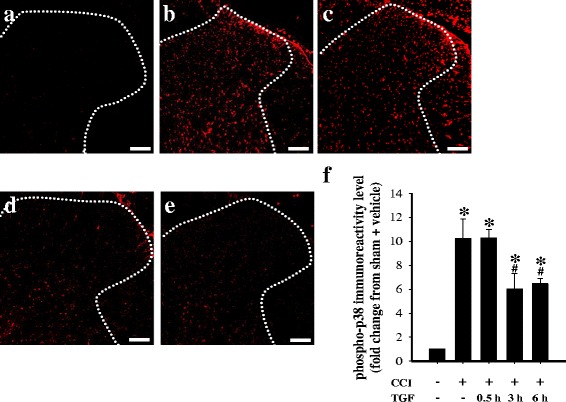
The effects of intrathecal (i.t.) transforming growth factor-β1 (TGF-β1) on chronic constriction injury (CCI)-induced upregulation of spinal phosphorylated (phospho)-p38 at 14 d post-surgery. Images represent cells labeled with phospho-p38 (*red*) in the spinal cord, obtained from the sham operated plus i.t. vehicle group (**a**), CCI plus i.t. vehicle group (**b**), and CCI plus i.t. TGF-β1 (5 ng) groups at 0.5 h (**c**), 3 h (**d**), and 6 h (**e**) after TGF-β1 injections. Quantification of the phospho-p38 immunoreactivity (**f**) demonstrates that TGF-β1 significantly inhibits CCI-induced upregulation of spinal phospho-p38. Each bar in (**f**) shows the mean ± standard error of the mean (SEM) from six rats per group. Scale bars: 100 μm for all images (**a**–**e**). *P < 0.05 compared to the sham operated plus vehicle group; #*P* < 0.05 compared to the CCI plus vehicle group

**Fig. 3:**
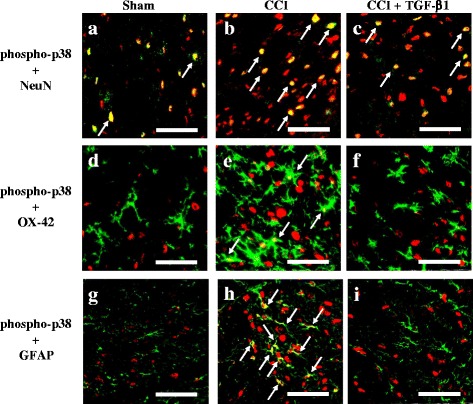
The effects of intrathecal (i.t.) transforming growth factor-β1 (TGF-β1) on chronic constriction injury (CCI)-induced upregulation of phosphorylated (phospho)-p38 in spinal neurons, microglia, and astrocytes at 14 d post-surgery. Merged images of double-immunofluorescence staining for phospho-p38 (*red*; **a**–**i**) with NeuN (neuronal-specific marker, *green*; **a**–**c**), OX-42 (microglial specific marker, *green*; **d**–**f**), and GFAP (astrocyte specific marker, *green*; **g**–**i**) in the lumbar spinal cord dorsal horn of the sham operated plus vehicle group (**a, d**, and **g**), CCI plus vehicle group (**b, e**, and **h**), and CCI plus TGF-β1 group (**c, f**, and **i**) at 3 h after i.t. injections. The results demonstrate that spinal phospho-p38 expression is localized to neurons, microglia, and astrocytes in the CCI plus vehicle group (*yellow*; *white arrow*), and was attenuated by i.t. TGF-β1. Scale bars: 50 μm for all images

Minimal and diffuse phospho-p38 immunoreactivity occurred in the ipsilateral dorsal horn of the lumbar spinal cord of the sham operated plus i.t. vehicle group (Fig. 2a). Phospho-p38 immunoreactivity increased in the ipsilateral spinal dorsal horn 14 d after CCI (Fig. 2b). At 0.5 h after TGF-β1 treatment, there was no significant inhibition of CCI-induced upregulation of phospho-p38 immunoreactivity (Fig. 2c). Quantification of the phospho-p38 immunoreactivity indicated that TGF-β1 significantly reversed CCI-induced upregulation of phospho-p38 immunoreactivity in the ipsilateral lumbar dorsal horn at 3 and 6 h after i.t. injections (Fig. 2f). We next examined the effects of i.t. injections of TGF-β1 on the cellular specificity of phospho-p38 expression in neuropathic rats, using double immunofluorescent staining. Neurons were labeled using anti-NeuN antibody (a neuron-specific nuclear marker) [24]. Microglia were visualized with anti-OX-42 antibody, which targets the microglial surface marker CD11b [24], and astrocytes were identified with anti-GFAP antibody, which labels the astrocytic intermediate filaments in the cytoplasm [24]. In the sham operated plus i.t. vehicle group, phospho-p38 was mainly localized to neurons (Fig. 3a). Upregulation of spinal phospho-p38 expression was observed in neurons (Fig. 3b), microglia (Fig. 3e), and astrocytes (Fig. 3h) at 14 d post-surgery in the CCI plus vehicle group, which was attenuated by TGF-β1 3 h after i.t. injections. In addition, CCI surgery activated microglia at 14 d post-surgery, as indicated by upregulation of OX-42 immunoreactivity and enlarged hypertrophic cell bodies with retraction of the cytoplasmic processes (Fig. 3e) [36] Fig. 3e). Furthermore, we observed activated astrocytes in rats with CCI at 14 d post-surgery, as indicated by upregulation of GFAP immunoreactivity and hypertrophied cell bodies with thickened processes (Fig. 3h) [37]. TGF-β1 markedly attenuated these effects in microglia and astrocytes.

### Effects of i.t. TGF-β1 on CCI-induced upregulation of spinal phospho-ERK expression

**Fig. 4:**
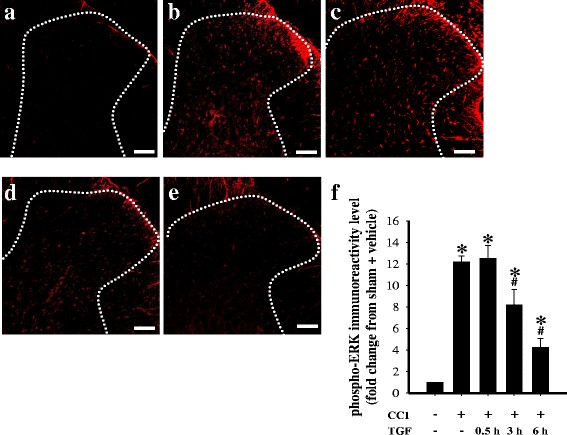
Effects of intrathecal (i.t.) transforming growth factor-β1 (TGF-β1) on chronic constriction injury (CCI)-induced upregulation of spinal phosphorylated extracellular signal-regulated kinase (phospho-ERK) at 14 d post-surgery. Images represent cells labeled with phospho-ERK (*red*) in the spinal cord, obtained from the sham operated plus i.t. vehicle group (**a**), CCI plus i.t. vehicle group (**b**), and CCI plus i.t. TGF-β1 (5 ng) groups at 0.5 h (**c**), 3 h (**d**), and 6 h (**e**) after TGF-β1 injections. Quantification of phospho-ERK immunoreactivity (**f**) demonstrates that TGF-β1 significantly inhibits CCI-induced upregulation of spinal phospho-ERK. Each bar in (**f**) shows the mean ± standard error of the mean (SEM) from six rats per group. Scale bars: 100 μm for all images (**a**–**e**). *P < 0.05 compared to the sham operated plus vehicle group; #*P* < 0.05 compared to the CCI plus vehicle group

**Fig. 5:**
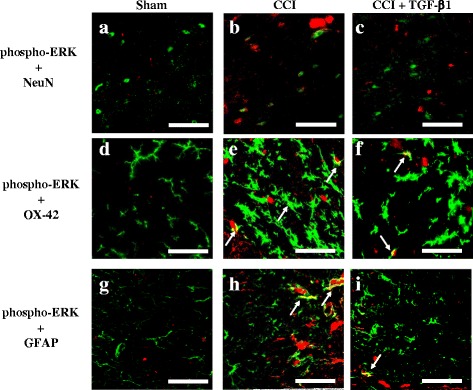
Effects of intrathecal (i.t.) transforming growth factor-β1 (TGF-β1) on chronic constriction injury (CCI)-induced upregulation of phosphorylated extracellular signal-regulated kinase (phospho-ERK) in spinal neurons, microglia, and astrocytes at 14 d post-surgery. Merged images of double-immunofluorescence staining for phospho-ERK (*red*; **a**–**i**) with NeuN (neuronal-specific marker, *green*; **a**–**c**), OX-42 (microglial specific marker, *green*; **d**–**f**), and GFAP (astrocyte specific marker, *green*; **g**–**i**) in the lumbar spinal cord dorsal horn, obtained from the sham operated plus vehicle group (**a, d**, and **g**), CCI plus vehicle group (**b, e**, and **h**), and CCI plus TGF-β1 group (**c, f**, and **i**) at 3 h after i.t. injections. The results demonstrate that spinal phospho-ERK expression is primarily localized to microglia and astrocytes in the CCI plus vehicle group (*yellow*; *white arrow*), and is attenuated by i.t. TGF-β1. Scale bars: 50 μm for all images

In the sham operated plus i.t. vehicle group, there was minimal and diffuse phospho-ERK immunoreactivity in the ipsilateral dorsal horn of the lumbar spinal cord (Fig. 4a). Phospho-ERK immunoreactivity significantly increased in the ipsilateral spinal cord of rats with CCI at 14 d post-surgery (Fig. 4b). At 0.5 h after i.t. injection, TGF-β1 treatment did not inhibit CCI-induced upregulation of phospho-ERK immunoreactivity (Fig. 4c). Quantification of phospho-ERK immunoreactivity demonstrated that i.t. TGF-β1 significantly inhibited CCI-induced upregulation of phospho-ERK immunoreactivity at 3 and 6 h after injection (Fig. 4f). We next studied the effects of i.t. TGF-β1 on the cellular specificity of phospho-ERK expression in neuropathic rats using double immunofluorescent staining. In the sham operated plus i.t. vehicle group, phospho-ERK was not localized to neurons (Fig. 5a), microglia (Fig. 5d), or astrocytes (Fig. 5g). In contrast, at 14 d post-surgery, upregulation of spinal phospho-ERK expression was observed in microglia (Fig. 5e) and astrocytes (Fig. 5h) of rats with CCI, which was markedly inhibited by TGF-β1 at 3 h after i.t. injection. In addition, at 14 d post-surgery, CCI resulted in both activated microglia (Fig. 5e) and astrocytes (Fig. 5h); these effects were also attenuated by i.t. TGF-β1.

## Discussion

### Summary of findings

In the present study, we found that i.t. TGF-β1 (5 ng) attenuated CCI-induced thermal hyperesthesia for as long as 6 h. Expression of phospho-p38 and phospho-ERK were upregulated in the spinal dorsal horn following CCI, and i.t. TGF-β1 reversed these effects. In addition, we found that both p38 and ERK are upregulated in activated microglia and astrocytes in the spinal dorsal horn following CCI. However, expression of phospho-p38 and phospho-ERK are more prominent in astrocytes. In addition, p38 occurs to a greater extent than ERK upregulation in neurons after CCI, and the expression of phospho-p38 in neurons is also downregulated after i.t. TGF-β1.

### The roles of spinal p38 and ERK in neuropathy

Clinical neuropathic pain syndromes are characterized by evoked pain, including hyperalgesia [38], which is represented as thermal hyperalgesia in the rat CCI model. Central sensitization in the spinal dorsal horn contributes to the hypersensitive pain behaviors associated with neuropathy [39]. Activation of microglia and astrocytes contribute to spinal neuroinflammation [40, 41] and accelerate central sensitization, as well as development and maintenance of neuropathic pain [11, 12]. Activated microglia and astrocytes in the spinal dorsal horn indicate elevated nociceptive states [18–20, 42–47], and CCI also resulted in activated microglia and astrocytes in the spinal cord at 14 d post-surgery. Phosphorylation of Tyr-182 and Thr-180 result in p38 activation [48]. Spinal phospho-p38 expression was only observed in neurons in the sham operated plus i.t. vehicle group. At 14 d post-surgery, upregulation of spinal phospho-p38 expression was observed in neurons, microglia, and astrocytes in rats with CCI. Moon et al. found that phospho-p38 staining was localized to neurons and astrocytes in the spinal cord of mice with CCI [49], and Gu et al. reported phospho-p38 in spinal microglia of rats with CCI [50]. The activated form of ERK is phosphorylated on both Thr-202 and Tyr-204 [51]. In the sham operated group, spinal phospho-ERK was not localized to neurons, microglia, or astrocytes. Upregulation of spinal phospho-ERK expression was observed in microglia and astrocytes of CCI rats, but not in neurons. However, the possibility that spinal neurons may also be a source of phospho-ERK expression cannot be excluded. Our double-immunostaining images further confirmed that spinal astrocytes are a major source of phospho-p38 and phospho-ERK upregulation in rats with CCI, compared to microglia. Our findings are consistent with a previous report that phospho-ERK is predominantly localized to astrocytes and minimally localized to microglia, but not localized to neurons in the spinal cord 21 d after spinal nerve ligation [24]. Our findings also support the hypothesis that spinal astrocytes contribute to maintaining neuropathic pain [13, 14, 24, 52].

### Contributions of p38 and ERK to the antinociceptive effects of TGF-β1

CCI evokes significant downregulation of spinal TGF-β1 protein in rats [8, 10, 53]. Similarly to previous results [8], we found that i.t. TGF-β1 attenuated CCI-induced thermal hyperesthesia in rats. Le et al. reported that TGF-β1 inhibits lipopolysaccharide (LPS)-induced phosphorylation of both p38 and ERK in a murine microglial cell line [54]. Similarly to this previous study, we found that TGF-β1 administration to rats reduced peripheral CCI-induced upregulation of phospho-p38 and phospho-ERK in activated spinal microglia. We also discovered that TGF-β1 reduced peripheral CCI-induced upregulation of phospho-p38 and phospho-ERK in activated rat spinal astrocytes. I.t. administration of a p38 inhibitor (SB203580) reduces neuropathic pain in animal models [22, 23, 25], and spinal nerve ligation-induced mechanical allodynia is attenuated by i.t. administration of the MAPK and ERK kinase (MEK; ERK kinase) inhibitor PD98059 [24]. At 0.5 h after i.t. injections, TGF-β1 did not inhibit CCI-induced upregulation of phospho-p38 or phospho-ERK immunoreactivity. However, the anti-hyperalgesic effects of TGF-β1 in rats with CCI reached the maximum %MPE at 0.5 h after administration. At 3 and 6 h after administration, TGF-β1 significantly suppressed CCI-induced upregulation of phospho-p38 and phospho-ERK immunoreactivity for the duration of time that the anti-hyperalgesic effects of TGF-β1 remained at > 50 % MPE. We previously reported that i.t. TGF-β1 (5 ng) reduced CCI-induced upregulation of spinal TNF-α [8] for the same duration of time that TGF-β1 inhibited CCI-induced phospho-p38 and phospho-ERK upregulation in the present study. These results are consistent with the finding that inhibiting activation of p38 and ERK block TNF-α gene expression in endotoxin-activated primary microglia and astrocytes [15]. Therefore, we suggest that inhibition of spinal phospho-p38 and phospho-ERK are primarily associated with the maintenance phase, but not with the development phase, of the antinociceptive effects of TGF-β1 during neuropathic pain.

## Conclusions

**Fig. 6:**
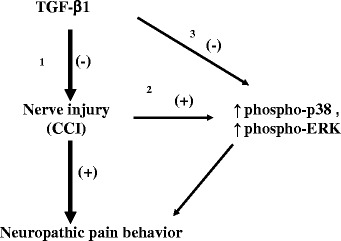
Schematic representation of possible mechanisms for the antinociceptive effects of intrathecal (i.t.) transforming growth factor-β1 (TGF-β1) during neuropathic pain. I.t. TGF-β1 may attenuate peripheral nerve injury-induced thermal hyperalgesia (*Pathway 1*). Upregulation of phosphorylated (phospho)-p38 and phosphorylated extracellular signal-regulated kinase (phospho-ERK) is a mechanism for nerve injury-induced pain (*Pathway 2*). The antinociceptive effects of i.t. TGF-β1 may occur via suppression of nerve injury-induced upregulation of phospho-p38 and phospho-ERK (*Pathway 3*)

Based on the present results and the findings of previous studies, we hypothesize that the antinociceptive effects of TGF-β1 are mediated by two different mechanisms (Fig. 6). First, TGF-β1 itself possesses antinociceptive effects, as demonstrated by the present study and our previous study [8]. Second, nerve injury may upregulate expression of phospho-p38 and phospho-ERK in spinal microglia and astrocytes, which may induce neuropathic pain behaviors. TGF-β1 may directly inhibit expression of phospho-p38 and phospho-ERK in microglia and astrocytes, which may reduce neuroinflammation, thereby attenuating neuropathic pain behavior in rats. Therefore, TGF-β1 is a promising therapeutic strategy for neuropathic pain.
